# Supplementary material for: A Likelihood Approach for Real-Time Calibration of Stochastic Compartmental Epidemic Models
Source: PLoS Comput Biol. 2017 Jan 17;13(1):e1005257. doi: 10.1371/journal.pcbi.1005257 (PMC5240920; doi:10.1371/journal.pcbi.1005257)
Supplement: S1 File — (TAR.GZ) [file pcbi.1005257.s014.tar.gz › HSPH_Online-SI-Revision/PF08/n100-mild/n100-mild_PF_table4.pdf]

1	R0	{1.24125, 3.38982}	{1.94061, 18.8309}	{{1.92069, 18.455}, {1.95015, 18.9767}}	{56.3433, 455.514}	inf	918.	17 175.6	{16 739.2, 17 335.}	1770.98	1.27204	newly	1628.	6275.17	{6178., 6372.}	285.453	0.585937	Reff	0.854936	0.94711	{0.937157, 0.953834}	10.7814	ar	34 926.	95 340.8	{93 749., 96 008.}	172.979
2	R0	{1.29554, 4.51878}	{1.89141, 16.93}	{{1.87436, 16.7823}, {1.91862, 16.9872}}	{45.994, 274.658}	inf	1374.	13 448.2	{13 195.8, 13 727.}	878.762	0.990569	newly	1875.	5381.68	{5263., 5525.}	187.023	0.457838	Reff	0.799646	0.869379	{0.861198, 0.875273}	8.72048	ar	42 828.	94 347.9	{93 486., 95 338.}	120.295
3	R0	{1.2443, 3.64292}	{2.08543, 19.4003}	{{1.8545, 16.456}, {2.12816, 19.8473}}	{67.5984, 432.549}	inf	748.	17 109.1	{14 651.5, 17 599.7}	2187.31	1.35858	newly	1275.	6064.	{5949., 6186.}	375.608	0.677201	Reff	0.825668	0.94283	{0.932486, 0.951791}	14.19	ar	37 087.	100 160.	{93 699., 101 607.}	170.069
4	R0	{1.38465, 3.43331}	{3., 20.}	{{3., 20.}, {3., 20.}}	{116.661, 482.528}	inf	741.	17 352.6	{17 120.7, 17 670.8}	2241.78	1.36951	newly	1149.	5456.63	{5338., 5592.}	374.903	0.676539	Reff	0.722441	0.400345	{0.395021, 0.407111}	44.5844	ar	49 511.	107 506.	{106 569., 108 312.}	117.136
5	R0	{1.25409, 4.1068}	{1.81457, 15.2051}	{{1.7916, 14.8684}, {1.83602, 15.4166}}	{44.6924, 270.242}	inf	1115.	12 843.	{12 635.5, 13 058.6}	1051.84	1.06136	newly	1641.	5660.13	{5547., 5762.}	244.92	0.537674	Reff	0.822708	0.840242	{0.835215, 0.851512}	2.13126	ar	38 571.	86 508.1	{85 906., 87 122.}	124.283
6	R0	{1.28821, 4.2016}	{1.97197, 17.7201}	{{1.95426, 17.3854}, {1.98802, 18.0945}}	{53.0779, 321.746}	inf	1013.	14 015.9	{13 533.8, 14 428.7}	1283.61	1.14086	newly	1399.	5311.81	{5186., 5426.}	279.686	0.579364	Reff	0.800326	0.819176	{0.804557, 0.837047}	2.35527	ar	41 468.	92 888.8	{92 029., 93 475.}	124.001
7	R0	{1.24369, 2.56007}	{2.77492, 20.}	{{2.7439, 20.}, {2.83168, 20.}}	{123.12, 681.229}	inf	442.	23 774.5	{23 438.6, 23 980.2}	5278.85	1.73067	newly	924.	7683.54	{7500., 7816.}	731.552	0.919843	Reff	0.781634	0.578478	{0.544423, 0.594568}	25.9913	ar	38 603.	99 781.1	{98 740., 101 008.}	158.48
8	R0	{1.232, 3.53942}	{1.88437, 17.7348}	{{1.86456, 17.3461}, {1.90915, 18.0213}}	{52.9521, 401.065}	inf	649.	15 498.5	{15 150.8, 15 877.8}	2288.06	1.37769	newly	1057.	5880.96	{5780., 5958.}	456.382	0.745327	Reff	0.831846	0.835332	{0.830316, 0.837848}	0.502412	ar	34 555.	94 498.3	{93 621., 95 830.}	173.472
9	R0	{1.36262, 3.85631}	{2.49424, 18.1115}	{{2.47897, 18.0063}, {2.51054, 18.2056}}	{83.0476, 369.658}	inf	951.	17 680.5	{17 555.7, 17 835.2}	1759.15	1.26931	newly	1368.	6237.38	{6129., 6343.}	355.949	0.658884	Reff	0.74132	0.539257	{0.534306, 0.542919}	27.2571	ar	48 410.	98 451.3	{98 006., 98 894.}	103.37
10	R0	{1.28702, 4.67968}	{1.85251, 18.179}	{{1.84508, 18.0672}, {1.86134, 18.313}}	{43.9382, 288.467}	inf	1183.	11 461.9	{11 363.2, 11 593.}	868.883	0.986255	newly	1557.	4261.74	{4194., 4338.}	173.715	0.437244	Reff	0.80682	0.841651	{0.835428, 0.846618}	4.31702	ar	41 092.	87 071.1	{86 421., 87 746.}	111.893
11	R0	{1.35752, 3.47129}	{2.08103, 16.9148}	{{2.07852, 16.8466}, {2.08405, 16.9652}}	{53.2964, 387.276}	inf	1161.	16 321.5	{16 267., 16 365.9}	1305.82	1.14793	newly	1963.	6404.37	{6320., 6491.}	226.254	0.513533	Reff	0.799932	0.777134	{0.773308, 0.780422}	2.85002	ar	44 669.	87 354.1	{87 076., 87 605.}	95.5588
12	R0	{1.35929, 5.83647}	{1.85628, 16.5167}	{{1.84543, 16.3628}, {1.87788, 16.7013}}	{36.5623, 182.991}	inf	1940.	12 378.9	{12 220.8, 12 532.4}	538.087	0.804774	newly	2036.	5005.49	{4925., 5079.}	145.849	0.390632	Reff	0.813535	0.800816	{0.797783, 0.803058}	1.5808	ar	46 715.	91 318.7	{90 721., 91 906.}	95.4804
13	R0	{1.28019, 3.29908}	{1.88996, 13.3828}	{{1.87316, 13.1296}, {1.90054, 13.6228}}	{47.6314, 305.651}	inf	878.	12 777.1	{12 466.4, 13 098.6}	1355.25	1.16284	newly	1478.	6188.01	{6060., 6319.}	318.675	0.621815	Reff	0.791618	0.736604	{0.724717, 0.744958}	6.9496	ar	40 941.	87 111.5	{86 397., 87 794.}	112.773
14	R0	{1.36661, 3.48318}	{3., 20.}	{{3., 20.}, {3., 20.}}	{119.521, 474.188}	inf	608.	17 206.1	{17 021.4, 17 378.1}	2729.94	1.45176	newly	929.	5455.95	{5355., 5570.}	487.293	0.768802	Reff	0.726053	0.458476	{0.441254, 0.471795}	36.8536	ar	48 672.	112 703.	{111 927., 113 617.}	131.556
15	R0	{1.35865, 3.84123}	{2.21007, 17.2809}	{{2.20563, 17.2011}, {2.21459, 17.4028}}	{62.6667, 349.88}	inf	1150.	17 127.8	{17 043.3, 17 264.4}	1389.37	1.173	newly	1651.	6480.64	{6377., 6567.}	292.528	0.593846	Reff	0.73825	0.692532	{0.686212, 0.699437}	6.19267	ar	48 598.	89 426.4	{89 170., 89 629.}	84.0126
16	R0	{1.22879, 3.98329}	{1.72854, 17.6351}	{{1.69989, 17.4055}, {1.78746, 17.8543}}	{40.6704, 342.726}	inf	1035.	11 688.5	{11 569.5, 11 825.7}	1029.32	1.0528	newly	1786.	4611.99	{4512., 4718.}	158.23	0.411948	Reff	0.876398	0.998175	{0.984898, 1.00713}	13.8952	ar	34 405.	84 111.4	{82 931., 85 941.}	144.474
17	R0	{1.34925, 3.64524}	{2.07902, 13.9602}	{{2.05556, 13.769}, {2.10663, 14.0691}}	{54.0869, 282.971}	inf	1239.	15 027.3	{14 844.4, 15 249.5}	1112.86	1.08379	newly	1851.	6963.42	{6804., 7153.}	276.198	0.575344	Reff	0.759911	0.721868	{0.709505, 0.740284}	5.00629	ar	46 871.	94 378.1	{93 807., 94 897.}	101.357
18	R0	{1.33054, 2.25275}	{3., 20.}	{{3., 20.}, {3., 20.}}	{125.472, 787.804}	inf	312.	23 558.	{23 462.9, 23 629.9}	7450.65	1.87798	newly	665.	7514.86	{7411., 7620.}	1030.05	1.05307	Reff	0.750025	0.500436	{0.498567, 0.501969}	33.2774	ar	44 336.	103 181.	{102 749., 103 551.}	132.724
19	R0	{1.28394, 3.69859}	{1.89508, 18.2077}	{{1.88243, 18.0849}, {1.91454, 18.2998}}	{47.5987, 392.287}	inf	963.	14 929.4	{14 744.3, 15 258.1}	1450.3	1.19038	newly	1523.	5632.51	{5515., 5726.}	269.83	0.567948	Reff	0.824444	0.934727	{0.921104, 0.947189}	13.3767	ar	39 558.	90 273.3	{89 429., 91 017.}	128.205
20	R0	{1.2883, 2.26493}	{3., 20.}	{{3., 20.}, {3., 20.}}	{132.865, 783.029}	inf	531.	26 774.1	{26 595.2, 26 978.1}	4942.21	1.70261	newly	1246.	8518.28	{8376., 8633.}	583.65	0.834803	Reff	0.78634	0.487897	{0.480127, 0.49693}	37.9534	ar	40 768.	107 165.	{106 597., 107 734.}	162.866
21	R0	{1.34098, 2.78621}	{2.10791, 12.6026}	{{2.03731, 12.5049}, {2.15935, 12.6642}}	{57.1915, 352.321}	inf	549.	14 977.5	{14 784.3, 15 337.1}	2628.15	1.43583	newly	1041.	7667.99	{7509., 7873.}	636.598	0.86716	Reff	0.763192	0.744674	{0.731327, 0.759194}	2.44986	ar	44 605.	102 654.	{101 796., 103 578.}	130.141
22	R0	{1.33783, 5.95911}	{1.78527, 17.3868}	{{1.71815, 16.5626}, {1.8334, 17.9406}}	{33.4452, 191.768}	inf	1990.	11 924.4	{11 275.5, 12 452.}	499.218	0.77718	newly	2166.	4644.65	{4457., 4795.}	114.434	0.331118	Reff	0.826485	0.863284	{0.826125, 0.890065}	4.51309	ar	45 418.	85 698.7	{82 945., 87 700.}	88.6888
23	R0	{1.33359, 3.3069}	{2.06497, 15.4998}	{{2.03888, 15.2059}, {2.0978, 15.6893}}	{54.8429, 368.712}	inf	1009.	17 047.6	{16 518.7, 17 470.8}	1589.56	1.22769	newly	1653.	7259.6	{7094., 7433.}	339.177	0.642567	Reff	0.751291	0.781634	{0.767908, 0.792193}	4.03874	ar	46 245.	94 102.5	{93 085., 95 211.}	103.487
24	R0	{1.30588, 2.56123}	{1.99756, 20.}	{{1.98925, 20.}, {2.00667, 20.}}	{52.9663, 680.875}	inf	372.	15 866.6	{15 764.5, 15 934.6}	4165.23	1.62991	newly	702.	5277.53	{5143., 5401.}	651.785	0.876013	Reff	0.762347	0.728676	{0.723553, 0.733891}	4.41667	ar	42 383.	97 245.8	{96 855., 97 634.}	129.445
25	R0	{1.29813, 4.51849}	{1.94117, 20.}	{{1.92415, 20.}, {1.95808, 20.}}	{49.5361, 342.626}	inf	1528.	14 946.1	{14 685.5, 15 201.5}	878.145	0.990357	newly	2037.	5125.06	{5003., 5228.}	151.598	0.40063	Reff	0.813343	0.912618	{0.899014, 0.932939}	12.2058	ar	43 371.	84 249.6	{83 673., 84 781.}	94.2532
26	R0	{1.33231, 3.99953}	{2.28037, 20.}	{{2.26586, 20.}, {2.30959, 20.}}	{71.1595, 400.059}	inf	1265.	17 333.2	{17 112., 17 602.3}	1270.22	1.13676	newly	1843.	5792.66	{5675., 5919.}	214.306	0.4973	Reff	0.789434	0.762952	{0.742148, 0.773422}	3.4074	ar	44 733.	98 245.2	{97 556., 98 737.}	119.626
27	R0	{1.30555, 4.61445}	{1.94989, 20.}	{{1.93162, 20.}, {1.96887, 20.}}	{49.3539, 333.421}	inf	1370.	15 157.1	{14 898.9, 15 375.7}	1006.36	1.04386	newly	1833.	5172.61	{5043., 5283.}	182.194	0.450486	Reff	0.814154	0.890332	{0.868451, 0.899086}	9.35671	ar	42 624.	85 147.7	{84 586., 85 777.}	99.7646
28	R0	{1.26819, 5.34969}	{1.82209, 18.1712}	{{1.8067, 17.4802}, {1.83922, 20.}}	{43.6761, 239.669}	inf	1478.	13 109.1	{12 550.7, 14 887.8}	786.95	0.946977	newly	1792.	4954.36	{4823., 5155.}	176.471	0.441523	Reff	0.858552	0.937163	{0.920085, 0.964978}	9.15626	ar	38 946.	88 663.3	{87 512., 90 733.}	127.657
29	R0	{1.19925, 3.00394}	{1.91822, 17.4823}	{{1.90536, 17.4269}, {1.92447, 17.5203}}	{59.9517, 481.979}	inf	570.	14 549.4	{14 470.3, 14 644.7}	2452.53	1.40697	newly	1093.	5754.02	{5662., 5853.}	426.443	0.721315	Reff	0.866182	0.975364	{0.972902, 0.978558}	12.605	ar	31 727.	92 245.3	{91 873., 92 631.}	190.747
30	R0	{1.35749, 3.75483}	{2.06993, 14.0016}	{{2.06549, 13.9422}, {2.07297, 14.038}}	{52.482, 272.896}	inf	1015.	14 353.1	{14 293.7, 14 411.6}	1314.1	1.15047	newly	1413.	6470.83	{6377., 6549.}	357.95	0.660793	Reff	0.732529	0.606806	{0.604068, 0.609117}	17.1628	ar	48 244.	96 163.7	{95 746., 96 543.}	99.3279
31	R0	{1.27131, 4.2161}	{1.8102, 15.8743}	{{1.79338, 15.6715}, {1.82083, 15.9789}}	{42.3889, 276.517}	inf	1215.	13 959.5	{13 714.5, 14 169.2}	1048.93	1.06021	newly	1803.	6049.03	{5947., 6158.}	235.498	0.525649	Reff	0.843883	0.956322	{0.948903, 0.964023}	13.324	ar	38 299.	91 026.3	{90 179., 91 676.}	137.673
32	R0	{1.31827, 6.18119}	{1.81748, 17.3668}	{{1.80906, 17.2235}, {1.83046, 17.5139}}	{37.8689, 180.963}	inf	1873.	12 064.	{11 985.9, 12 191.8}	544.1	0.808896	newly	1902.	4688.33	{4601., 4764.}	146.495	0.391764	Reff	0.818804	0.844656	{0.833266, 0.852289}	3.17275	ar	45 262.	88 303.6	{87 716., 89 174.}	95.0944
33	R0	{1.20398, 1.35167}	{3., 20.}	{{3., 20.}, {3., 20.}}	{149.174, 1379.65}	inf	90.	27 466.	{27 365.6, 27 571.}	30 417.8	2.48455	newly	298.	8802.63	{8690., 8904.}	2853.9	1.47037	Reff	0.806895	0.537324	{0.532558, 0.541662}	33.4085	ar	33 329.	103 224.	{102 737., 103 726.}	209.712
34	R0	{1.32776, 3.146}	{2.82875, 20.}	{{2.7777, 20.}, {2.84144, 20.}}	{113.047, 535.728}	inf	466.	18 948.8	{18 776.7, 19 227.4}	3966.26	1.60917	newly	799.	6010.58	{5900., 6128.}	652.263	0.87632	Reff	0.745949	0.444861	{0.436854, 0.456948}	40.3631	ar	44 872.	97 890.2	{97 247., 98 444.}	118.154
35	R0	{1.279, 1.11336}	{3., 20.}	{{3., 20.}, {3., 20.}}	{134.558, 1696.36}	inf	15.	29 008.5	{28 915., 29 117.2}	193 290.	3.28643	newly	47.	9433.96	{9305., 9548.}	19 972.3	2.30257	Reff	0.754597	0.635955	{0.631945, 0.640719}	15.7225	ar	41 033.	101 816.	{101 404., 102 204.}	148.132
36	R0	{1.20029, 1.94717}	{2.75337, 20.}	{{2.74766, 20.}, {2.76154, 20.}}	{129.392, 927.132}	inf	338.	26 442.3	{26 295.4, 26 619.9}	7723.17	1.89338	newly	910.	8697.13	{8578., 8816.}	855.729	0.98031	Reff	0.82688	0.698758	{0.691296, 0.70292}	15.4946	ar	33 234.	99 118.9	{98 804., 99 587.}	198.245
37	R0	{1.33216, 3.69685}	{2.07208, 17.7595}	{{2.05991, 17.5486}, {2.1154, 17.8897}}	{55.5426, 380.395}	inf	945.	15 744.6	{15 506.7, 15 852.4}	1566.1	1.22167	newly	1507.	5877.72	{5782., 5975.}	290.028	0.591053	Reff	0.770335	0.754204	{0.725532, 0.765647}	2.09403	ar	45 235.	89 273.	{88 758., 89 863.}	97.3538
38	R0	{1.25434, 2.04314}	{3., 20.}	{{3., 20.}, {3., 20.}}	{139.17, 878.885}	inf	246.	25 557.9	{25 288., 25 790.9}	10 289.4	2.01658	newly	612.	8137.81	{7998., 8279.}	1229.71	1.12372	Reff	0.777829	0.494385	{0.481141, 0.505807}	36.4404	ar	38 662.	100 983.	{100 424., 101 487.}	161.196
39	R0	{1.27466, 3.0741}	{2.35875, 17.051}	{{2.35174, 16.9259}, {2.3705, 17.1414}}	{85.0492, 454.666}	inf	499.	17 227.6	{17 089.9, 17 366.3}	3352.42	1.53811	newly	937.	6452.59	{6349., 6542.}	588.644	0.837965	Reff	0.783495	0.574986	{0.571151, 0.578921}	26.6127	ar	40 181.	103 113.	{102 706., 103 500.}	156.621
40	R0	{1.33045, 2.68154}	{3., 20.}	{{3., 20.}, {3., 20.}}	{125.488, 645.84}	inf	318.	21 372.5	{21 279.4, 21 472.6}	6620.9	1.82743	newly	616.	6689.74	{6598., 6777.}	985.997	1.0358	Reff	0.729978	0.376209	{0.372959, 0.37923}	48.463	ar	45 951.	104 320.	{103 370., 105 002.}	127.025
41	R0	{1.37003, 2.83984}	{3., 20.}	{{3., 20.}, {3., 20.}}	{118.973, 604.265}	inf	516.	23 683.	{23 662.3, 23 793.8}	4489.73	1.66177	newly	894.	7491.46	{7388., 7614.}	737.971	0.923194	Reff	0.718704	0.441615	{0.437021, 0.442671}	38.554	ar	48 926.	101 351.	{101 052., 101 611.}	107.152
42	R0	{1.3003, 2.94885}	{2.59131, 18.4736}	{{2.57748, 18.4233}, {2.60868, 18.5223}}	{99.2857, 526.467}	inf	576.	20 389.9	{20 258.7, 20 533.2}	3439.92	1.54899	newly	1090.	7061.03	{6947., 7161.}	547.801	0.811411	Reff	0.746177	0.548314	{0.54507, 0.551196}	26.5169	ar	44 237.	100 394.	{100 036., 100 789.}	126.946
43	R0	{1.33756, 2.92653}	{3., 20.}	{{3., 20.}, {3., 20.}}	{124.289, 583.403}	inf	776.	22 929.7	{22 828.9, 23 040.4}	2854.85	1.47053	newly	1372.	7306.93	{7208., 7407.}	432.575	0.726357	Reff	0.730161	0.487124	{0.483637, 0.491328}	33.2853	ar	47 342.	101 945.	{101 528., 102 356.}	115.338
44	R0	{1.35801, 2.92276}	{3., 20.}	{{3., 20.}, {3., 20.}}	{120.911, 584.285}	inf	436.	22 979.7	{22 879.5, 23 098.9}	5170.56	1.72185	newly	782.	7222.79	{7121., 7328.}	823.63	0.965476	Reff	0.722081	0.402875	{0.397302, 0.407919}	44.2064	ar	47 940.	104 681.	{103 940., 105 447.}	118.358
45	R0	{1.31342, 4.02649}	{1.81616, 14.6026}	{{1.8064, 14.4463}, {1.82374, 14.6905}}	{38.2773, 262.663}	inf	1233.	12 861.3	{12 720.1, 13 013.9}	943.093	1.0183	newly	1772.	5908.	{5795., 6014.}	233.409	0.522939	Reff	0.7963	0.852091	{0.846562, 0.858568}	7.02305	ar	42 995.	85 991.6	{85 328., 86 541.}	100.004
46	R0	{1.37919, 3.75889}	{2.1741, 14.1889}	{{2.14468, 13.8933}, {2.20542, 14.3841}}	{57.6357, 277.475}	inf	1312.	16 181.1	{15 796.6, 16 538.}	1133.31	1.091	newly	1998.	7399.64	{7159., 7647.}	270.352	0.568486	Reff	0.747438	0.727049	{0.714802, 0.737068}	2.72784	ar	49 157.	96 496.2	{95 489., 97 621.}	96.3021
47	R0	{1.37458, 3.56117}	{2.83498, 20.}	{{2.75628, 20.}, {2.86798, 20.}}	{106.244, 461.613}	inf	838.	20 742.7	{20 540.4, 21 089.2}	2375.27	1.39359	newly	1272.	6607.02	{6471., 6741.}	419.42	0.715464	Reff	0.732981	0.484896	{0.469004, 0.504664}	33.8461	ar	48 478.	101 014.	{100 090., 101 699.}	108.371
48	R0	{1.33393, 3.78296}	{1.99349, 20.}	{{1.98356, 20.}, {1.99729, 20.}}	{49.445, 428.687}	inf	1093.	16 050.5	{15 883.1, 16 111.5}	1368.48	1.16686	newly	1686.	5516.41	{5435., 5607.}	227.189	0.514763	Reff	0.779095	0.930095	{0.926582, 0.933015}	19.3815	ar	44 885.	85 792.9	{85 415., 86 185.}	91.1393
49	R0	{1.24918, 2.39046}	{2.00981, 13.6813}	{{2.00014, 13.5917}, {2.02045, 13.7479}}	{60.8905, 472.33}	inf	342.	13 998.4	{13 771.3, 14 141.4}	3993.09	1.61203	newly	806.	6669.82	{6544., 6782.}	727.521	0.917739	Reff	0.817189	0.753433	{0.745246, 0.759328}	7.80181	ar	35 969.	92 547.4	{92 087., 92 994.}	157.298
50	R0	{1.28281, 2.3032}	{3., 18.6274}	{{3., 18.5367}, {3., 18.7547}}	{133.862, 708.761}	inf	333.	17 634.8	{17 456.3, 17 912.3}	5195.73	1.7239	newly	744.	5942.65	{5839., 6045.}	698.743	0.90237	Reff	0.782989	0.440607	{0.436522, 0.44413}	43.7276	ar	40 223.	102 537.	{102 059., 103 187.}	154.922
